# Green Inhibition of Corrosion of Aluminium Alloy 5083 by *Artemisia annua* L. Extract in Artificial Seawater

**DOI:** 10.3390/molecules28072898

**Published:** 2023-03-23

**Authors:** Gloria Zlatić, Ivana Martinović, Zora Pilić, Andrea Paut, Ivana Mitar, Ante Prkić, Dušan Čulum

**Affiliations:** 1 Department of Chemistry, Faculty of Science and Education, University of Mostar, 88 000 Mostar, Bosnia and Herzegovina; 2 Faculty of Chemistry and Technology, University of Split, Ruđer Bošković 35, 21000 Split, Croatia; 3 Faculty of Science, University of Split, Ruđer Bošković 33, 21000 Split, Croatia; 4 Department of Chemistry, Faculty of Science, University of Sarajevo, 71 000 Sarajevo, Bosnia and Herzegovina

**Keywords:** natural antioxidants, plant extracts, corrosion, inhibition efficiency, marine environment, electrochemical methods

## Abstract

Plant extracts are increasingly being examined in the corrosion inhibition of metal and alloys in various environments due to their potent antioxidant properties. The use of *Artemisia annua* L. aqueous extract (AAE) as an aluminium alloy 5083 (ALA) corrosion inhibitor in artificial seawater (ASW) was investigated using electrochemical tests and spectroscopy tools, while the active biocompounds found in AAE were analyzed using high-performance liquid chromatography (HPLC). Electrochemical results showed that AAE acts as an anodic inhibitor through the physisorption (Δ*G* ≈ –16.33 kJ mol*^−^*^1^) of extract molecules on the ALA surface, thus reducing the active sites for the dissolution of the alloy in ASW. Fourier-transform infrared spectra confirmed that phenolic acids found in AAE formed the surface layer that protects ALA against the corrosive marine environment, while HPLC analysis confirmed that the main phytoconstituents of AAE were chlorogenic acid and caffeic acid. The inhibition action of phenolic acids and their derivatives found in the AAE was based on the physisorption of caffeic acid on the ALA surface, which improved physicochemical properties of the barrier film and/or conversion of Al^3+^ to elemental aluminium by phenolic acids as reducens, which slowed down the diffusion rate of Al^3+^ to or from the ALA surfaces. The protective effect of the surface layer formed in the presence of AAE against ASW was also confirmed by inductively coupled plasma–optical emission spectrometry (ICP-OES) whereby the measured concentration of Al ions after 1 h of immersion of ALA in the pure ASW was 15.30 μg L^−1^ cm^−2^, while after the addition of 1 g L^−1^ AAE, the concentration was 3.09 μg L^−1^ cm^−2^.

## 1. Introduction

More than 5% of oxygen that is used in the respiration processes of living functional cells turns to reactive oxygen species (ROS) [1]. Exceeded levels of ROS disrupt the transduction of chemical or physical signal pathways in biological redox systems, which may lead to a dysfunction or collapse of a biological cell [2,3,4]. Oxidation of basic biomolecules and cell structures is also associated with aging processes [5,6,7] and reduction of nutritional value and quality of food products [8,9]. This problem led to the discovery and examination of natural antioxidants, along with the synthesis of new ones. Synthetic and natural antioxidants act as free radical acceptors by scavenging mechanisms or by inhibiting processes of lipid peroxidation and/or formation of ROS which prolong cell life; therefore, they are applied in the healthcare, cosmetic, and food industries [10,11,12,13,14]. Synthetic antioxidants such as butylated hydroxyanisole and butylated hydroxytoluene have been reported to harm human health [15,16,17], thus many industries are focusing on the development and the use of naturally occurring antioxidants and their derivatives [18,19].

Nature provides numerous renewable sources of natural antioxidants, both high and low molecular weight compounds such as enzymes, proteins, vitamins, and polyphenols that manifest different chemical and physical properties [20,21]. Plants are the most substantial source of natural antioxidants, while the most abundant naturally occurring antioxidants in the plant kingdom are polyphenolics, which are found in all parts of the plants [22]. These include phenolic acids, flavonoids, lignans, stilbenes, and tannins [23]. The antioxidant activity of some phenolics is described by either proton donation mechanism, single-electron transfer, or a combination of both [24,25]. In the presence of ionic metals, polyphenols can act as chelating agents and coordinate with metal ions through π-electrons of unsaturated bonds or through lone pairs of electrons found in hydroxy, carbonyl, and other functional groups attached to the aromatic ring [26,27,28,29].

Since the stability of alloys used in oxygen-rich environments is dependent on the physicochemical properties of protective passivation film formed on alloy surface, which prevents them from attacks by corrosive agents [30,31], plant antioxidants are also being examined as renewable, green corrosion inhibitors as they can cause complexation of the antioxidant with the metal surface, which may increase the resistance of the protective film and reduce the chemical reactivity of the metal/alloy surface exposed to atmospheric or dissolved oxygen in different environments [32,33,34,35,36,37,38,39,40,41]. The adsorption mechanisms of plant antioxidants on the metal surfaces obey Langmuir, Temkin, or Freundlich adsorption isotherms, where the physisorption of the extract mainly occurs with inhibition efficiency between 50–80% [34,39]. The significant antioxidant activity of plant extracts is not restricted to a particular family and is mostly attributed to the synergetic effect of active constituents found in the extracts [20]. Our recent study suggests that *Artemisia annua* L. harvested in its naturally occurring habitat in Herzegovina exhibits high antioxidant activity and has a high content of phenolics, flavonoids, and tannins [42]. *Artemisia annua* L. is a wildflower known in traditional medicine for centuries [43,44,45,46]. Since *A. annua* has been used in traditional medicine in Bosnia and Herzegovina for a long time, it was very interesting to investigate the anti-corrosion behavior of locally grown *A. annua*. To the best of our knowledge, there have not been records about using *A. annua* extracts from Bosnia and Herzegovina as corrosion inhibitors. Moreover, it is well known fact that geographic origin significantly influences the phytochemical content of plants. Therefore, a different impact on corrosion can be expected. Hence, our intention was to check the possibility of using the extracts of locally grown *A. annua* for corrosion inhibition. It belongs to the *Compositae* family and is integrated into The Plant List database under a TICA source [47]. Previous studies suggest that the chemical composition of the aqueous plant extract mainly consisted of sesquiterpenoids and other compounds such as flavonoids and mono-feruloyl-quinic, mono-caffeoyl-, di-caffeoyl-, and di-feruloyl-quinic acids [48,49]. The presence of polyphenols with active groups in *A. annua* aqueous extract makes it a suitable natural antioxidant for examination of its anticorrosive activity in different electrolytes. Therefore, the present work is focused on examining the potential inhibitory effect of *A. annua* extract on the corrosion of aluminium alloy 5083 in a simulated marine environment. From the corrosion point of view, the marine environment represents a challenging medium for metal and alloy applications. The most important factors that determine the rate of corrosion reactions of metals and alloys in seawater are temperature, salinity, pH, the concentration of dissolved gasses, and the presence of microorganisms [50,51,52]. In the presence of chlorides and/or microbes, growth and repair of naturally formed oxide film on an aluminium surface are retarded, and if there are not sufficient amounts of oxygen or other oxidizing agents in the examined medium, the barrier film might break in weak spots or inhomogeneities which results in the localized dissolution of aluminium [50,53,54,55].

The low density and high metal strength of aluminium made it applicable in various industrial applications [51,52,53], but because of its susceptibility to localized galvanic processes, it has been widely replaced by aluminium alloys [56,57]. Aluminium–magnesium alloys, 5000 series, are used in many marine applications because of their versatile benefits of strength, weldability, and high corrosion resistance [58,59,60]. The electrochemical behavior of aluminium alloy 5083 (ALA) in artificial seawater (ASW) without and with the addition of *A. annua* extract (AAE) at different concentrations was examined using Cyclic Voltammetry (CV), Potentiodynamic Polarization (PP), and Electrochemical Impedance Spectroscopy (EIS) measurements. The aqueous extraction of plant material was executed at room temperature to deliberately bypass the use of excess energy and solvents that generate a huge amount of waste by-products in the plant extract preparation processes. The main phytoconstituents found in the studied plant extract were identified with the high-performance liquid chromatography (HPLC) system. Corrosion parameters obtained from electrochemical results were then used in determining the adsorption mechanism of extract molecules on aluminium alloy surfaces. The composition and the stability of the surface film formed on aluminium alloy during immersion in artificial seawater without and with the addition of *A. annua* were examined by Fourier transform infrared spectroscopy (FTIR) and inductively coupled plasma–optical emission spectrometry (ICP-OES).

## 2. Results

### 2.1. High-Performance Liquid Chromatography

**Figure 1:**
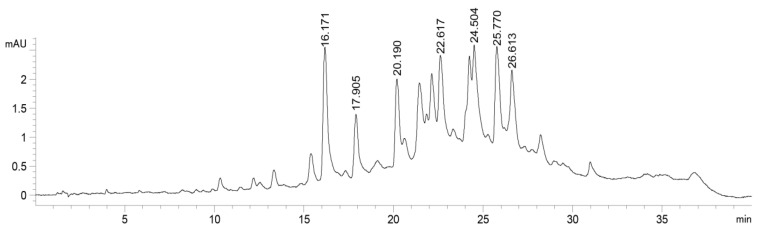
The HPLC chromatograph (325 nm) of the aqueous extract of *A. annua*.

High-performance liquid chromatography (HPLC) analysis revealed the presence of phenolic compounds in AAE (Figure 1). Taking into consideration of the error bar (<10 ppm), two active compounds were identified: chlorogenic acid and caffeic acid (Table 1). The identification of phenolic compounds detected in the samples was performed by comparing the retention times (*R_t_*) and UV spectra of the compounds in the samples with the *R_t_* and UV spectra of the standards recorded at the same conditions. The concentration of chlorogenic acid and caffeic acid found in the AAE (40 mg mL^−1^) was 28.64 ± 0.55 ppm and 7.70 ± 0.08 ppm, respectively (Table 1). As for earlier chromatographic investigations, some of the most abundant phytochemicals in aqueous *A. annua* extract were caffeic and quinic acid derivatives [44,46,48,49].

### 2.2. Electrochemical Tests

#### 2.2.1. Cyclic Voltammetry

**Figure 2:**
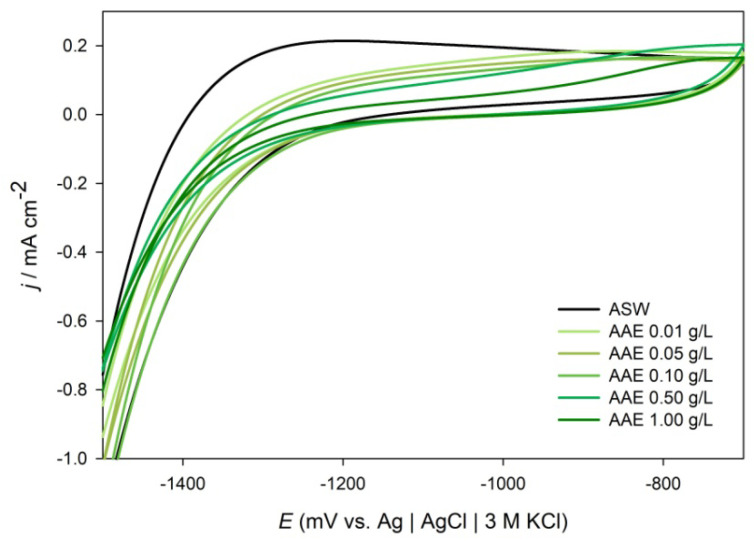
Cyclic voltammograms for the aluminium alloy 5083 recorded in ASW without and with the addition of different concentrations of *Artemisia annua* L. extract (AAE, shown in figure); *ν* = 30 mV s^−1^.

The cyclic voltammograms of the aluminium alloy 5083 recorded in ASW without and with the addition of AAE (0.01–1.0 g L^−1^) at a scan rate of 30 mV s^−1^ are presented in Figure 2. The voltammogram of aluminium alloy 5083 recorded in pure ASW showed the current shoulder at around −1.3 V, which can be associated with the accumulation of anions and cations on the working electrode’s interface that initially do not have enough electric field strength for movement. This initial phase, or the “incubation time”, forego the growth of the barrier film of the aluminium alloys [61]. The accumulation of Al(III) ions on the electrode surface was followed by a current plateau that was reached around −1.1 V and corresponds to the growth of the surface film with a constant electric field [61,62].

The addition of AAE to the ASW solution led to a decrease in the anodic current densities in the potential region that corresponds to the accumulation of Al(III) ions. During the addition of different concentrations of AAE (0.01–1.0 g L^−1^), anodic peaks were observed in the potential range between −1.00 and −0.70 V that represent the formation of Al_2_O_3_. The formation of Al_2_O_3_ on the aluminium surface is irreversible, so no peak in the cathodic scan was recorded [33,61,62,63]. As presented in Figure 2, the total charges used in the oxidation processes, *Q_A_* decreased as the AAE concentration increased. *Q_A_* values were determined by integrating the anodic part of the cyclic voltammograms and then used to calculate the surface coverage of the ALA surface by AAE molecules (Equation (2)) and the thickness of the film formed (Equation (4)). The inhibition efficiency was calculated according to Equation (3). Table 2 gives surface coverage, inhibition efficiency, and the film thickness of ALA in examined solutions.

As presented in Table 2, coverage of the ALA surface by AAE molecules increased from 0.18 for 0.01 g L^−1^ AAE to 0.786 for 1.00 g L^−1^ AAE, while the film thickness calculated from CV data showed a decrease from 1.00 nm for pure ASW to 0.21 nm for 1.00 g L^−1^ AAE. Obtained parameters also showed that the addition of 1.00 g L^−1^ AAE protected the ALA surface in ASW with an inhibition efficiency of 78.6%.

#### 2.2.2. Electrochemical Impedance Spectroscopy

**Figure 3:**
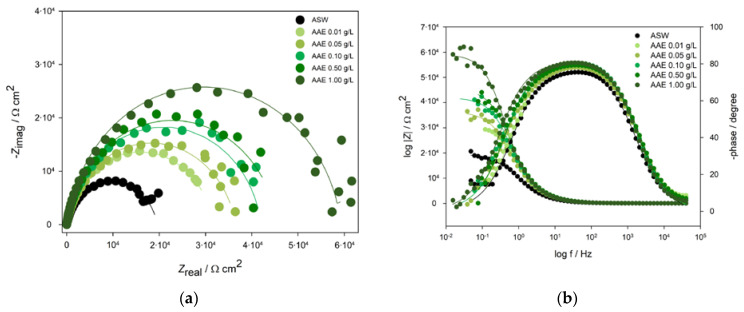
Nyquist (**a**) and Bode (**b**) plots of aluminium 5083 alloy recorded in ASW without and with the addition of different concentrations of *Artemisia annua* L. extract (AAE, shown in figure). Solid lines represent simulated data (χ^2^ ≤ 10^−4^, with acceptable error of fitting elements ±5%).

The inhibitor activity of *Artemisia annua* L. extract (AAE) on aluminium 5083 alloy (ALA) corrosion in a simulated marine environment was further examined by impedance measurements at the open circuit potential. Impedance spectra, presented by Nyquist and Bode plots, are given in Figure 3. Nyquist plots of impedance spectra recorded after 1 h immersion of Al 5083 samples in ASW without and with the addition of AAE showed a clearly defined capacitive loop. It is evident that with the increase of AAE concentration, the radius of the capacitive semi-circle also increases, indicating an increase in the resistance of the surface layer formed on the ALA surface in the presence of AAE.

The results of impedance spectra for ALA recorded in ASW without and with the addition of different concentrations of AAE were described by one-time constant model [*R*(*R*_1_*CPE*_1_)], where *R* represents resistance, and *CPE* the constant phase element with the impedance equal to the following expression:

(1) $$Z_{CPE}\left(\omega \right)={\left[Q{\left(j\omega \right)}^{n}\right]}^{-1}$$

where *Q* is a constant, *ω*—angular frequency, and *n* represents the power of *CPE* that describes the porosity and roughness of the surface and can have values in the range from −1 to 1.

Impedance spectra for ALA recorded in ASW without and with the addition of AAE (0.01–1.00 g L^−1^) consisted of the resistance of the solution, *R* connected in series with the charge transfer resistance, *R*_1_, and double layer capacitance *CPE*_1_ combined in parallel. The double layer capacitance formed on the Al alloy 5083 surface in ASW without and with the addition of different concentrations of AAE, and corresponding charge transfer resistances were therefore associated with the fast charge transfer process in the alloy dissolution reaction in the ASW. Values of EEC elements are given in Table 3.

As seen from Table 3, the addition of 0.01 g L^−1^ AAE to ASW led to an increase in the charge transfer resistance from 19.5 kΩ cm^−2^ to 31.6 kΩ cm^−2^ and a decrease in the capacitance of the electrochemical double layer from 14.9·10^−6^ to 9.4·10^−6^ Ω^−1^s^n^ cm^−2^, which may refer to the adsorption of AAE molecules on the ALA surface. The value of the exponent of the *CPE*_1_ *n*_1_ ≈ 0.9 (0.01 g L^−1^ AAE) indicated a homogeneous coverage of the ALA surface with AAE molecules. Furthermore, the same trend in the increase of charge transfer resistance was observed during the addition of a higher concentration of AAE, where the initial *R*_1_ value of 19.5 kΩ cm^−2^ in the pure ASW increased up to 36.5, 41.9, 45.6, and 59.3 kΩ cm^−2^ for AAE concentrations of 0.05, 0.10, 0.50, and 1.00 g L^−1^, respectively. Because of the role of mass transport and charge transfer through interfacial boundaries, *R*_1_ was considered in the evaluation of inhibition efficiency (*η*). Accordingly, *η* values that were calculated using Equations (3) and (5) ranged from 38.2–67.0% where the maximum inhibition efficiency of 67.0% was recorded at the highest AAE concentration (1.00 g L^−1^). During the addition of AAE, a decrease in the *CPE*_1_ values were observed, while the *n*_1_ ≈ 0.9 values indicated homogeneity of the surface layer.

#### 2.2.3. Potentiodynamic Polarization

**Figure 4:**
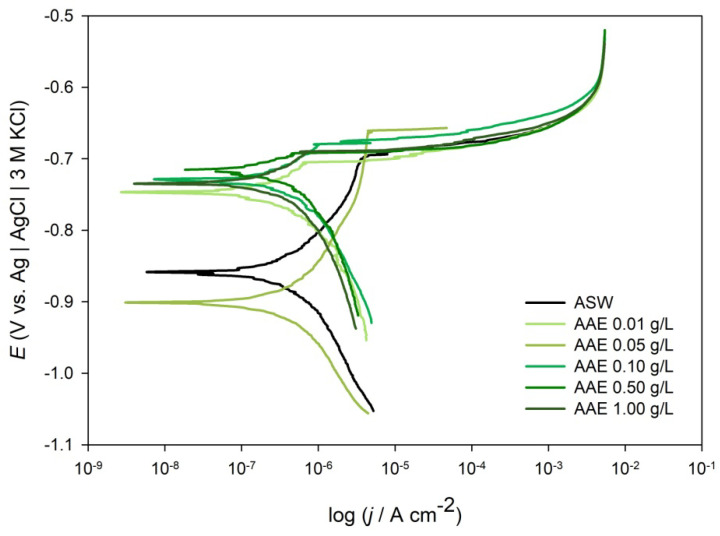
Potentiodynamic polarization curves for the Al alloy 5083 in ASW, containing different concentrations of *Artemisia annua* L. extract (AAE, shown in figure); *ν* = 0.5 mV s^−1^.

Figure 4 shows the potentiodynamic polarization (PP) curves of Al alloy 5083 in ASW solution without and with the addition of different concentrations of AAE. The polarization curves of Al alloy 5083 in ASW without and with the addition of AAE showed similar shape, except that the curves were shifted toward lower current densities when the AAE was added to the ASW at different concentrations. The addition of AAE to ASW also shifted the corrosion potential of ALA positively. The kinetic parameters obtained from PP curves are presented in Table 4 along with the calculated *θ* (Equation (6)) and *η* values (Equation (3)).

As can be seen from Table 4, the addition of AAE to ASW induced a positive shift of the free corrosion potential to the anodic direction. Observed anodic shifts toward the positive values for ALA recorded in ASW at 0.01, 0.10, 0.50, and 1.00 g L^−1^ AAE concentration were 124, 132, 143, and 124 mV, respectively. Since the corrosion potential shift was more than +85 mV, *A. annua* can be classified as an anodic inhibitor [64,65,66,67]. Current densities, *j*_corr_, obtained from PP curves decreased with the increase of AAE concentration, where *j*_corr_ for 1.00 g L^−1^ AAE decreased three times compared to the *j*_corr_ recorded in pure ASW. As presented in Table 4, addition of AAE to ASW from 0.01–1.00 g L^−1^ increased surface coverage from 0.175 to 0.665 where the highest inhibition efficiency of 66.7% was recorded for 1.00 g L^−1^ AAE.

### 2.3. Adsorption Mechanism

**Figure 5:**
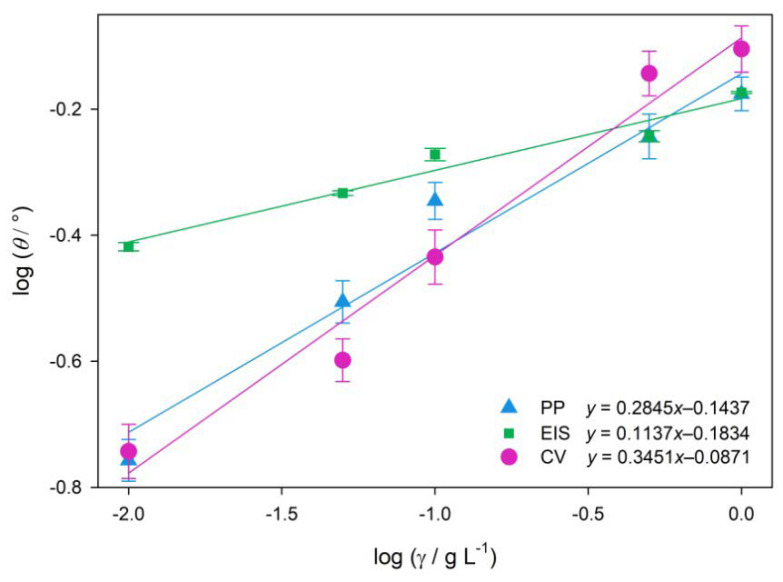
Freundlich isotherms of adsorption of *A. annua* extracts on Al alloy 5083 surfaces in ASW.

Electrochemical results showed that extract molecules of *Artemisia annua* L. prevent ALA dissolution in ASW by adsorbing on the active sites of alloy surfaces. The mechanisms of interaction between AAE molecules and the ALA coupon surface can be defined using adsorption isotherms. Although some researchers draw attention that the adsorption mechanism of plant extracts cannot be determined by the standard free energy of adsorption since the molecular mass of the extract molecules is unknown [68,69], other researchers suggested that the adsorption mechanism between a metal surface and plant extract molecules can be obtained using the adsorption constant, *K* values [70]. In this work, the degree of surface coverage (*θ*) for the examined *A. annua* extract concentrations in ASW were calculated from CV, EIS, and PP data using Equations (2), (5) and (6), respectively. The obtained electrochemical results were then graphically tested to fit different isotherms, where linear correlation was achieved using the Freundlich adsorption isotherm. The results are presented in Figure 5.

The adsorption constant, *K*, and the Gibbs free energy, Δ*G*, determined from PP, EIS and CV data using Equation (7), were as follows: *K* = 0.718 and Δ*G* = −16.30 kJ mol^−1^ (PP), *K* = 0.655 and Δ*G* = −16.07 kJ mol^−1^ (EIS), *K* = 0.818 and Δ*G* = −16.62 kJ mol^−1^ (CV).

### 2.4. Surface Film Characterization

**Figure 6:**
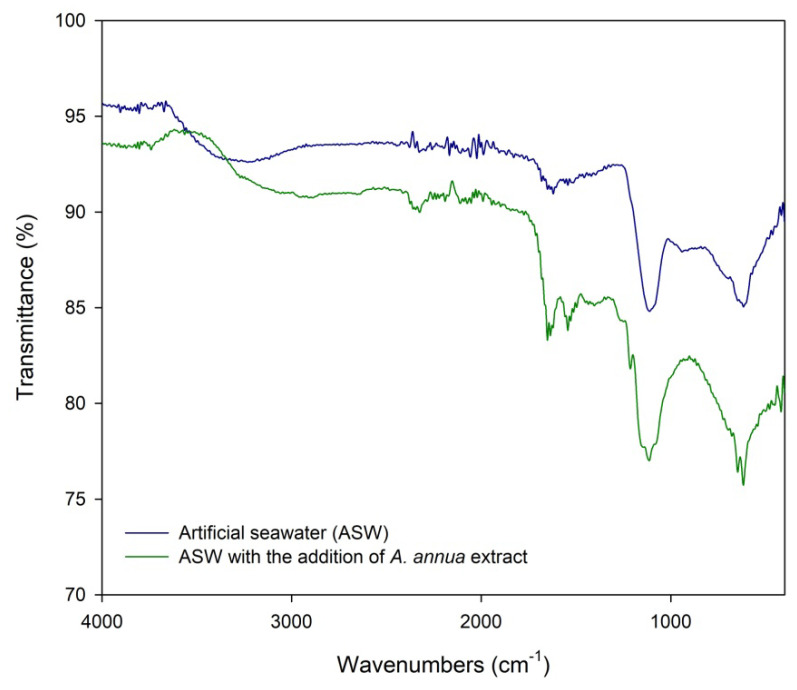
FTIR spectra of the Al alloy 5083 surface after 24 h immersion in ASW without and with the addition of AAE (1 g L^−1^).

The FTIR spectra of Al alloy 5083 coupons that had been immersed in ASW without and with the addition of AAE for 24 h are shown in Figure 6.

The broad peak that appeared in the range of 2900–3600 cm^−1^ was attributed to the O–H stretching of water molecules adsorbed on the ALA surface that was immersed in pure ASW. The band that appeared between 2600–3400 cm^−1^ on the samples immersed in the inhibited medium could be attributed to the hydroxyl stretching of water or carboxylic acid molecules. A small broad peak appearing at 1630 cm^−1^ for coupons that were immersed in ASW was assigned to the O–H bending modes of H_2_O molecules that were adsorbed on the ALA surface. In the same region, the addition of AAE to ASW led to the appearance of two sharp peaks at 1540 and 1640 cm^−1^ which could correspond to C=O band stretching [71,72]. The shifts in frequencies indicated that AAE has coordinated with Al^3+^ on the ALA surface, probably through oxygen atoms of carbonyl or hydroxyl groups found in the structures of polyphenols extracted from *A. annua*. By analyzing the FTIR spectra of ALA surface treated with AAE (Figure 6), it can be concluded that most corrosion inhibition of AAE can be attributed to the adsorption of carboxylic acids as well as phenols on the alloy surface. This is in accordance with the chromatographic investigation of AAE, where chlorogenic and caffeic acid were detected (Figure 1, Table 1) in the studied extract. The presence of vibration bands at 1100–400 cm^−1^ corresponds to the vibration of O–Al–O bonds in Al_2_O_3_ [73,74,75,76] that was formed on the ALA surface during 24 h immersion in ASW and probably served as a protective barrier from the corrosive environment. The strong sharp bands that appeared between 1000 and 1200 cm^−1^ for both examining samples were assigned to γ-Al_2_O_3_ [76,77,78] and α-Al_2_O_3_ [79,80]. The other intense peak appearing between 500 and 800 cm^−1^ in both samples was attributed to α-Al_2_O_3_ [81,82,83,84,85]. Two small sharp peaks that appeared at 1120 and 1200 cm^−1^ on the samples treated with the AAE could correspond to functional groups responsible for the adsorption of AAE molecules on the ALA surface during the 24 h immersion test, which resulted in the improvement of the structural properties of the protective film formed on the ALA surface in the presence of AAE [86,87,88,89].

The concentration of the dissolved Al^3+^ ions in the ASW was determined with the help of ICP-OES, both in the presence and absence of 1 g L^−1^ AAE after 1 h and 24 h immersion tests. Results of the concentration of Al^3+^ ions expressed in μg L*^−^*^1^ cm^−2^ are reported in Table 5, along with the corresponding corrosion rates and inhibition efficiencies.

As shown in Table 5, the addition of AAE (1 g L^−1^) during 1 h immersion tests of the ALA in ASW led to a decrease in corrosion rates from 0.76 to 0.15 μg cm^−2^ h^−1^. Additionally, good corrosion inhibition of ALA in ASW was obtained during the immersion tests for a duration of 1 h, which was in agreement with the inhibition efficiencies obtained from the electrochemical data. Immersion tests with a duration of 24 h led to a decrease in *η* values from 79.73% (1 h) to 13.51%.

## 3. Discussion

It is well known that chloride ions present in the marine environment can cause pitting attacks and breakage of the passive Al_2_O_3_ film on aluminium and its alloys’ surfaces, so material loses corrosive stability which can manifest in different ways [90]. The aim of this study was to determine if *Artemisia annua* L. is a suitable green, raw material for corrosion inhibition of Al alloy 5083 (ALA) in a simulated marine environment. The cyclic voltammograms showed that *A. annua* reduces anodic charges on the ALA coupon surfaces and decreases the surface film thickness from 1.00 nm (for ASW) to 0.21 nm (for 1 g L^−1^ AAE, Table 2). Since the magnitude of *j*_corr_ represents the speed of the reaction [91], results indicated that the rate of dissolution of Al alloy 5083 was lower when the AAE was added to ASW, probably as a consequence of the adsorption of AAE molecules on the ALA surface that blocked the active sites of the material. This was supported by the calculated values of surface coverage, where during the addition of 1 g L^−1^ AAE to ASW, 0.786 of the ALA surface was covered with AAE molecules (Table 2). As evident from Nyquist plots (Figure 3a), the radius of the semi-circle increased with the increase of AAE concentration, which was associated with the adsorption of AAE molecules on the ALA surface that led to the improvement in the resistance of the surface layer. In addition, Bode plots (Figure 3b) showed an increase in the impedance at the low-frequency region with an increase in AAE concentration, while in the middle-frequency range, a maximum phase angle of −82° was recorded for the highest AAE concentration. A decrease in *CPE*_1_ values (from 14.9–9.9·10^−6^ Ω^−1^ s^n^ cm^−2^) and an increase in *R*_1_ (from 19.5 to 59.3 kΩ cm^−2^) for 1.00 g L^−1^ AAE compared to pure ASW were probably caused by a decrease in the local dielectric constant of the surface layer due to adsorption of AAE molecules on the ALA surface which inhibited further corrosion, where *n*_2_ ≈ 0.9 indicated a homogeneous coverage of the ALA surface with AAE molecules (Table 3). The incommensurate change in *CPE*_1_ values with the increase in AAE concentration (Table 3) could be due to the substitution of water by AAE molecules and/or changes in the hydrophobicity of the ALA surface that may occur after treatment of metal surfaces with the plant extracts [70,92]. PP data revealed the displacement of corrosion potential to values higher than +85 mV with the addition of AAE compared to pure ASW, indicating that *A. annua* acts as an anodic inhibitor by adsorbing on active sites of metal and blocking the dissolution of ALA.

**Figure 7:**
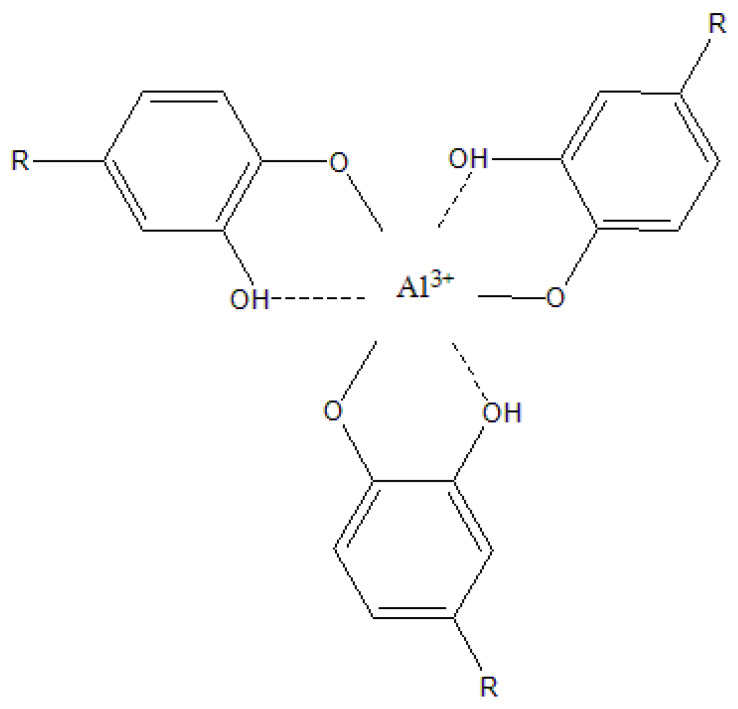
Chemical structure of Al(III) complex with caffeic acid as a ligand, R: CH=CHCOOH or CH=CHCOO^−^.

All electrochemical tests were used in the assessment of the adsorption mechanism of the extract on the ALA surface. Chemisorptions involve charge transfer or sharing between material surfaces and extract molecules that result in coordinative bonding. In this case, Gibbs free energy of adsorption has a value of −40 kJ mol^−1^ or higher. On the other hand, physisorption with values of Δ*G* = −20 kJ mol^−1^ or less describes electrostatic interactions between the charged extract molecules and charged metal surfaces [35,93]. CV, EIS, and PP data showed that adsorption of the AAE molecules on the ALA surface obeys the Freundlich adsorption isotherm, with the physisorption mechanism (Δ*G* ≈ –16.33 kJ mol^−1^). However, recent criticism of the use of adsorption isotherms to distinguish between chemisorption and physisorption of plant extract compounds on metal surfaces summons the inability of present electrochemical methods to deduce from the complex chemical composition of plant extracts which compound is responsible for inhibitory effect [94]. Even though numerous experimental data showed a correlation between plant extract concentration and the surface charge on metal [32,33,34,35,36,37,38,39,40,41,65,95], the adsorption mechanism should not be deduced only from electrochemical data, especially because physisorption energy scales with the size of the molecule, whereas high calculated Gibbs free energy values might refer to physisorption of larger organic molecules, due to the establishment of multiple weak electrostatic interactions with the metal surface [96]. The HPLC analysis showed that AAE consisted of different types of active biocompounds (Figure 1), where chlorogenic and caffeic acid were identified as some of the main phytoconstituents of AAE (Table 1). The adsorption of these phenolic compounds on the ALA surface was confirmed by FTIR spectra of coupons exposed to ASW without and with the addition of 1 g L^−1^ AAE which showed vibration modes of hydroxyl and carbonyl functional groups (Figure 6). The broad peak between 2600–3400 cm^−1^ and two sharp peaks at 1540 and 1640 cm^−1^ were attributed to carboxyl and hydroxyl band vibrations [71,72]. The latter could confirm that organic molecules that were attracted to the positively charged metal surfaces were phenolic acids and/or their derivatives, such as caffeic and chlorogenic acid, which were identified by the HPLC analysis. The vibration modes of other functional groups that could furthermore confirm the identity of phenolic acid adsorbed on the ALA surface could be overlapping with the vibration modes of the main corrosion products of both samples: γ-Al_2_O_3_ and α-Al_2_O_3_. Our recent study showed that the oxidation of phenolic components responsible for the antioxidant activity of the aqueous extract of *A. annua* was reversible and that one of the main components of the examined plant extract was caffeic acid [42]. Earlier studies reported that the main organic components of *A. annua* aqueous extract were caffeic and feruloyl-quinic acid derivative [48,49], while this study confirmed that small molecules, such as chlorogenic and caffeic acid, readily diffused to the aqueous phase during 3 h maceration at room temperature. In the presence of AAE, the phenolic acids and their derivatives are also attracted to the ALA surfaces, through electrostatic forces between lone electron pairs of oxygen atoms of carbonyl or hydroxyl groups and the charged surfaces of the working electrode. In the examined media, caffeic acid could have acted as a chelating agent and coordinated with the charged aluminium surface through oxygen atoms of hydroxyl groups at positions 3 and 4 of the phenyl ring (Figure 7), which consequently resulted in the formation of a more resistant surface film.

**Figure 8:**
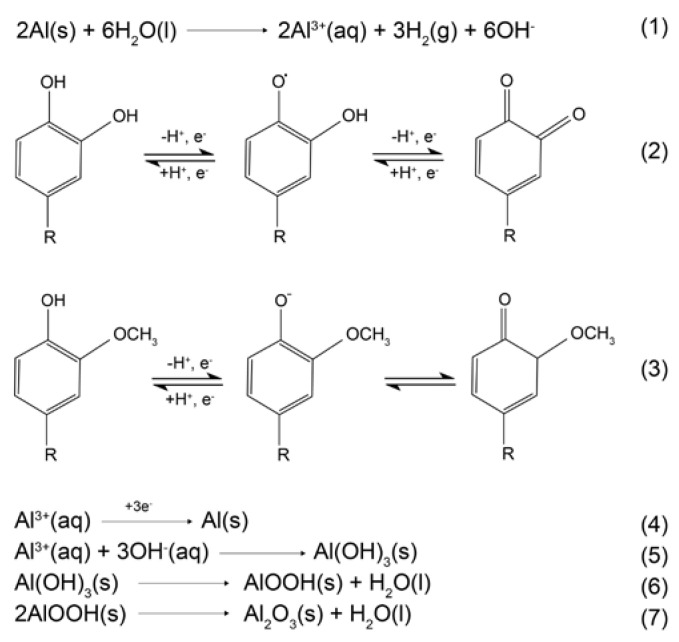
Proposed mechanism of the Al dissolution rate decrease by phenolic acids and the formation of Al_2_O_3_, R: CH=CHCOOH or CH=CHCOO^−^.

A decrease in the diffusion rate of Al^3+^ ions to or from the Al alloy 5083 surfaces could also be due to the reduction of Al ions by caffeic acid or other phenolic acids, as proposed in the mechanism of the formation of Al_2_O_3_ (Figure 8). The mechanism in the following text was proposed and adapted to the specific case according to the literature sources [97,98,99], where more detailed descriptions of all products obtained can be found.

As shown in Figure 8, during the anodic dissolution of Al (1), the aluminium cations attract phenolic acids, so either the electrostatic interaction between caffeic acid and Al^3+^ were formed (Figure 7), and/or the interaction included electron exchange between aluminium cations and phenolic acids found in the AAE. It is well known that the reversible oxidation of caffeic acid leads to quinone via semiquinone form (2) [94,96], while the reversible oxidation of ferulic acid is a result of deprotonation of a hydroxyl group on the position 4 at the aromatic ring (3) [95]. Since the decrease in anodic current densities observed at cyclic voltammograms recorded with the addition of AAE (Figure 2) indicated that extract molecules inhibited the accumulation of Al^3+^ at the electrode surface, while the PP results classified AAE as an anodic inhibitor, it is possible that during the oxidation of phenolic acids, aluminium ions served as electron acceptors, which resulted in a reduction of Al^3+^ to elemental aluminium (4). As electrochemical double-layer formation continues, unreduced aluminium cations precipitate in the form of Al(OH)_3_ (5). The aluminium hydroxide is then dehydrogenated to AlOOH-oxyhydroxide (6) and finally converted to aluminium oxide (7), γ-Al_2_O_3_ and α-Al_2_O_3_.

Good protective ability of the surface layer in the presence of AAE was confirmed by ICP-OES. When examining film properties by ICP-OES, a shortcoming of this method should be considered, as it only measures ions that diffused to the electrolyte, without measuring what’s left in the corrosion products [95]. Here, ICP-OES was used as a confirmatory test of the stability of the film formed on the ALA surface during immersion in ASW with and without the addition of 1 g L^−1^ AAE. The inhibition efficiency obtained from ICP-OES data after short immersion tests (1 h) was in accordance with values obtained from electrochemical results, except that no significant difference in dissolved ions was observed during a longer immersion test (24 h) of ALA in ASW without and with the addition of AAE. A decrease in inhibition efficiency from 79.73 to 13.51% for 1 h and 24 h immersion tests (Table 5), respectively, suggests that weak electrostatic interactions were established between AAE molecules and the metal surface; therefore, future work should include an additional examination and/or optimization of the stability of the layer formed on the ALA surface in the presence of AAE.

## 4. Materials and Methods

### 4.1. The Working Electrode and Working Electrolyte

Samples of aluminium alloy 5083 (ALA) with 20 × 20 × 3 mm dimensions were purchased from Frankstahl d.o.o. (Vitez, Bosnia and Herzegovina). Chemical composition of the working electrode, provided by the manufacturer’s declaration, was as follows: (%) 0.1809 Si, 0.3380 Fe, 0.0329 Cu, 0.4882 Mn, 4.3489 Mg, 0.0882 Cr, 0.0209 Zn, 0.0211 Ti, 94.4405 Al. Before each experiment, the surface of the working electrode was mechanically abraded with 420–1200 granulation SiC grinding paper and then washed with distilled water, ultrasonically degreased in ethanol, and thoroughly rinsed with ultrapure water (Millipore Simplicity UV Water Purification System, Burlington, MA, USA) and working solution, respectively. The same steps were followed for electrochemical tests, except that the additional step of the removal of air-formed surface oxides by the polarization of the electrode surface for 120 s at −1.60 V vs. Ag|AgCl|3 M KCl was added as the last step of the procedure. The working electrolyte for all conducted experiments was artificial seawater (ASW) prepared in ultrapure water with the following chemical composition: (g L^−1^): 4.1575 Na_2_SO_4_, 11.1211 MgCl_2_ × 6H_2_O, 0.79023 KCl, 1.5877 CaCl_2_ × 2H_2_O, 24.9772 NaCl, 0.0587 NaHCO_3_ [100].

### 4.2. Collection and Preparation of Plant Material

Aerial parts of the stem of sweet wormwood, *Artemisia annua* L., were collected in the flowering stage during September 2020 in the southeastern part of Bosnia and Herzegovina (Stolac). Botanical identification of the plant material was verified with the help of dr. sc. Anđelka Lasić, systematic botanist of the Department of Biology, Faculty of Science and Education, University of Mostar. Freshly harvested plant material was dried in a dark, ventilated room at temperatures below 40 °C. The final product was stored in the dark at 20 °C. A stock solution of *Artemisia annua* extract (AAE) was prepared as follows: 1.00 g of dried grounded sample of *A. annua* was mixed with 1.00 L of ASW and set aside in the dark with occasional stirring of the mixture. After 3 h, the sample was filtered through a 0.45 μm filter paper and diluted up to a final volume of 1.00 L.

In order to determine the adsorption mechanism of AAE on the ALA surface in ASW, the initial concentration of 1.00 g L^−1^ was diluted up to four more concentrations (g L^−1^: 0.01, 0.05, 0.10, 0.50). For chromatographic analysis, the sample was prepared as follows: 100 mL of 3.5% NaCl solution was added to 4 g of dried, grounded plant material, and the mixture was left at room temperature for 3 h, manually stirred from time to time, and then filtered. Before analysis, the sample was diluted with ultrapure water in a 1:100 ratio.

### 4.3. High-Performance Liquid Chromatography Analysis

In order to identify phytochemicals in the *Artemisia annua* L. aqueous extract (AAE) obtained at room temperature, the sample was prepared as described in Section 4.2. The qualitative and quantitative analysis of phytochemicals was carried out using an Agilent 1290 Infinity high-performance liquid chromatography (HPLC) system (Agilent Technologies, Santa Clara, CA, USA) coupled to a Diode Array Detector (G4212A) with binary pump (G4220A) and Thermostatted Column Compartment (G1316C). Chromatographic separation of the chemical constituents in AAE was performed on a ZORBAX C18 column (5 µm, 15 cm × 4.66 mm, Agilent Technologies, Santa Clara, CA, USA) at a flow rate of 1 mL min^−1^, at a temperature of 25 °C. The mobile phases consisted of methanol (A) and 0.1% formic acid (B), and the separation of components was conducted by applying the following gradients: 0–15 min, 0–25% A, 15–30 min, 25–60%, 30–35 min, 60–70%, 35–40 min, 70–0% A. The injection volume in the HPLC system was 20 μL, and the UV–Vis detection was performed in the 270–370 nm wavelength range [101]. All applied standards and solvents for HPLC analysis were of the highest purity grade available and purchased from Sigma-Aldrich Co. (Munich, Germany). Calibration curves based on HPLC analysis used to quantify phytochemicals in AAE can be found in Supplementary Materials. Control of the experiment and data analyses were executed with the help of Agilent Chemstation software (version LTS 01.11).

### 4.4. Electrochemical Tests

The electrochemical behavior of the ALA in ASW with and without the addition of AAE was investigated using the electrochemical techniques of cyclic voltammetry (CV), potentiodynamic polarization (PP), and electrochemical impedance spectroscopy (EIS). All electrochemical measurements were performed in ASW without and with the addition of AAE (g L^−1^: 0.01, 0.05, 0.10, 0.50, 1.00) on Autolab PGSTAT320N potentiostat (Metrohm, Herisau, Switzerland). All experiments were conducted in a standard three-electrode electrochemical cell (Model K0235 Flat Cell), where the exposed surface of the working electrode was 1 cm^2^, platinum in the form of a mesh served as the counter electrode, and the reference electrode to which all potentials in this work are referred was Ag|AgCl|3 M KCl. The results of PP, EIS, and CV measurements were analyzed with Nova software, version 1.5.

#### 4.4.1. Cyclic Voltammetry

Cyclic voltammograms were recorded in a potential range from −1600 to −700 mV, with the scan rate of 30 mV s^−1^. Analysis of cyclic voltammograms of ALA in ASW, without and with the presence of AAE taken at the scan rate of 30 mV s^−1^, gave total anodic charges *Q_A_* which were used in the determination of surface coverage (Equation (2)) and inhibition efficiency (Equation (3)) using the following equations:

(2) $$\theta =\frac{Q_{A}-Q_{A}\left(\mathrm{AAE}\right)}{Q_{A}}$$

(3) η=θ·100

Obtained *Q_A_* and *Q_A_* (AAE) values were also used to calculate the thickness, *d* (Equation (4)) of a surface layer formed from corrosion products on the ALA surface in examined solutions.

(4) $$d=\left(\frac{M}{\rho zF}\right)\frac{Q_{A}}{\sigma }$$

where *M* represents the molar mass of Al_2_O_3_, *ρ*—density of Al_2_O_3_ (*M*/*ρ* = 31.80 cm^3^ mol^−1^), *z*—number of electrons, *F*—Faraday constant, and *σ* represents the surface roughness factor (*σ* = 2).

#### 4.4.2. Electrochemical Impedance Spectroscopy

Electrochemical impedance spectroscopy (EIS) measurements were performed at the open circuit potential (OCP), which was stabilized and monitored for 1 h, after which the alternating voltage of 10 mV was applied in the frequency range of 5 mHz to 10 kHz. Results of EIS spectra were fitted to equivalent electrical circuits (EEC), where the charge transfer resistance, *R*_1_, was extrapolated from obtained Nyquist plots in the examined systems (*R*_1_—ASW with the addition of AAE at different concentrations, *R*_1_^0^ ASW without AAE) and then used to calculate surface coverage (Equation (5)) and inhibition efficiency (Equation (3)).

(5) $$\theta =\frac{R_{1}-R_{1}^{0}}{R_{1}}$$

#### 4.4.3. Potentiodynamic Polarization

EIS was followed by PP measurements, where PP curves were recorded from −200 mV in the cathodic direction vs. corrosion potential, *E*_corr_, to 200 mV in anodic direction at a scan rate of 0.5 mV s^−1^. Mathematical analysis of Tafel plots recorded in ASW with and without the addition of AAE, gave basic electrochemical polarization parameters, where obtained corrosion current densities, *j*_corr_, were used to calculate the surface coverage (Equation (6)) and inhibition efficiency (Equation (3)).

(6) $$\theta =\frac{j_{\mathrm{corr}}^{0}-j_{\mathrm{corr}}}{j_{\mathrm{corr}}^{0}}$$

where *j*_corr_^0^ was recorded in pure ASW and *j*_corr_ in ASW with the addition of AAE at different concentrations.

### 4.5. Adsorption Mechanism

The surface coverage, *θ*, for different concentrations of AAE, calculated using CV, EIS, and PP data, were used to plot the relationship between the *θ* and the concentration of the AAE molecules that were adsorbed on the ALA surface. After testing various adsorption isotherms, linearity was achieved using the Freundlich adsorption isotherm, where Equation (7) was used to calculate the equilibrium adsorption constant, *K*, and adsorption intensity, *n*.

(7) log θ=log K−n log γ

Gibbs free energy of adsorption, Δ*G*^0^ (Equation (8)), was calculated using equilibrium adsorption constants obtained from CV, EIS, and PP data to determine if the adsorption of AAE molecules that occurred on the ALA surface in ASW was rather physisorption or chemisorption [35].

(8) $$\Delta G^{0}=-RT\;\ln\;\left[1000\left({g\;L}^{-1}\right)\cdot K\left(g^{-1}L\right)\right]$$

where 1000 represents the mass concentration of water in the solution (g L^−1^), *R*—universal gas constant, *T*—absolute temperature.

### 4.6. Surface Film Characterization

The efficiency of AAE as an inhibitor was also examined by elemental determination of aluminium as a trace element in a working electrolyte after immersion of ALA samples. Before analysis, the tested material was prepared as described in Section 4.3, and then immersed in 50 mL ASW solution without and with the addition of AAE (1.0 g L^−1^) for 1 h and 24 h. The concentration of dissolved Al^3+^ ions in the working electrolyte after immersion of tested material in ASW without and with the addition of AAE for 1 h and 24 h was measured with inductively coupled plasma optical emission spectrometry (ICP–OES, iCAP 6500 Duo, Thermo Scientific, Cambridge, UK). Calibration for the analyses was performed using standard stock solution (Multi–Element Plasma Standard Solution 4, Specture^®^, Alfa Aesar, John Mutthey Company, London, UK). Obtained results were used in the determination of corrosion rates (Equation (9)) of ALA in ASW without and with the addition of AAE.

(9) $$\nu =\frac{W}{At}$$

where *W* is the mass of dissolved ions in μg, *A* is the surface area of the electrodes exposed to working electrolyte in cm^2^, and *t* is the duration of immersion in reference [95].

Calculated *ν* values were then used in the determination of inhibition efficiency with the following expression [95]:

(10) $$\eta =\frac{\nu^{0}-\nu }{\nu^{0}}\cdot 100$$

where *ν*^0^ and *ν* represent the corrosion rate of ALA specimens without and with the addition of AAE, respectively.

After 24 h immersion tests ALA coupons were dehydrated successively with ethanol solutions (50%, 75%, and 96% by volume) for 10 min each. Chemical composition of the ALA surface after 24 h immersion in 50 mL ASW without and with the addition of AAE was analyzed using Fourier transform infrared spectroscopy, FTIR (IRAffinity-1S, Shimadzu, Tokyo, Japan) equipped with diamond and optical crystal sampling plate (GladiATR10, Shimadzu, Tokyo, Japan) in the range of 4000–400 cm^−1^.

## 5. Conclusions

This study revealed that *A. annua* extract molecules have good corrosion inhibition properties for aluminium alloy type 5083 against the corrosive marine environment. HPLC analysis showed that chlorogenic and caffeic acids were some of the main phytoconstituents of AAE. Electrochemical results indicated that *A. annua* acts as an anodic inhibitor by adsorption of extract molecules on the ALA surface, thus reducing the active sites for dissolution of the tested material. The main corrosion products of ALA in ASW were γ-Al_2_O_3_, and α-Al_2_O_3_. The protective effect of AAE was attributed to the physisorption (Δ*G* ≈ −16.33 kJ mol^−1^) of phenolic acids such as chlorogenic, ferulic, and caffeic acids on the ALA surface that resulted in formation of a thinner, more resistant barrier film, as confirmed by obtained FTIR spectra. The inhibition action was also due to a reduction in diffused Al^3+^ to elemental aluminium with phenolic acids as a reducing agent. The protective effect of the film against ASW was also confirmed by ICP-OES.

## Figures and Tables

**Table 1:** Retention time (*R_t_*), molecular formula, and the concentration of the phenolic compounds in the studied *A. annua* aqueous extract.

*R_t_* (min)	Compound	Molecular Formula	*γ* (ppm)	LOD (ppm)	LOQ (ppm)
16.171	Chlorogenic acid	C_16_H_18_O_9_	28.64 ± 0.55	4.05	12.28
17.905	Caffeic acid	C_9_H_8_O_4_	7.70 ± 0.08	0.58	1.75

**Table 2:** Anodic charges, film thickness, surface coverage, and inhibition efficiency for the Al 5083 alloy in ASW without and with the addition of different concentration of *Artemisia annua* L. extract (AAE).

*γ* (g L^−1^)	*Q_A_* (µC cm^−2^)	*d* (nm)	*θ*	*η* (%)
0	36.4	0.99	−	−
0.01	29.8	0.81	0.181	18.1
0.05	27.2	0.75	0.252	25.2
0.10	23.0	0.63	0.368	36.8
0.50	10.2	0.28	0.719	71.9
1.00	7.8	0.21	0.786	78.6

**Table 3:** Impedance parameters of Al 5083 alloy in ASW without and with the addition of different concentrations of *Artemisia annua* L. extract (AAE).

*γ*	*R*	*R*_1_	*CPE*_1_ × 10^−6^	*n*_1_	*θ*	*η*
g L^−1^	Ω cm^−2^	kΩ cm^−2^	Ω^−1^s^n^ cm^−2^			%
0	19.3	19.53	14.9	0.879	–	–
0.01	19.6	31.59	9.4	0.905	0.382	38.2
0.05	19.6	36.47	11.4	0.887	0.464	46.4
0.10	18.3	41.97	9.9	0.912	0.534	53.4
0.50	19.6	45.57	9.3	0.902	0.571	57.1
1.00	19.3	59.29	9.9	0.912	0.670	67.0

**Table 4:** Kinetic parameters from polarization measurements and calculated values of surface coverage, *θ*, and inhibition efficiency, *η*, for the Al 5083 alloy in ASW without and with the addition of different concentrations of *Artemisia annua* L. extract (AAE).

*γ*	*E*_corr_	*j*_corr_	*β*_a_	*β*_c_	*θ*	*η*
g L^−1^	V	μA cm^−2^	mV dec^−1^	mV dec^−1^		%
0	–0.859	0.673	0.197	0.238	–	–
0.01	–0.735	0.556	0.167	0.228	0.175	17.5
0.05	–0.800	0.463	0.141	0.171	0.312	31.2
0.10	–0.727	0.369	0.107	0.124	0.451	45.1
0.50	–0.716	0.289	0.081	0.145	0.571	57.1
1.00	–0.735	0.224	0.077	0.102	0.667	66.7

**Table 5:** Effect of the AAE on corrosion of aluminium alloy 5083 in ASW as determined by ICP-OES.

AAE Concentration (g L^−1^)	Dissolved Al^3+^ (μg L^−1^ cm^−2^)	Corrosion Rate (μg cm^−2^ h^−1^)	*η* (%)
0 (1 h)	15.30	0.765	–
1.00 (1 h)	3.09	0.155	79.73
0 (24 h)	17.85	0.037	–
1.00 (24 h)	15.45	0.032	13.51

## Data Availability

Not applicable.

## Supplementary Materials

The following supporting information can be downloaded at: https://www.mdpi.com/article/10.3390/molecules28072898/s1, Table S1: Concentration of the phenolic compounds found in the studied *A. annua* aqueous extract (AAE) with corresponding Limit of Detection (LOD), and Limit of Quantitation (LOQ) values based on HPLC analysis; Figure S1: Calibration curve based on HPLC analysis used to quantify chlorogenic acid in AAE; Figure S2: Calibration curve based on HPLC analysis used to quantify caffeic acid in AAE.
