# Supplementary material for: Green Inhibition of Corrosion of Aluminium Alloy 5083 by Artemisia annua L. Extract in Artificial Seawater
Source: Molecules. 2023 Mar 23;28(7):2898. doi: 10.3390/molecules28072898 (PMC10095662; doi:10.3390/molecules28072898)
Supplement: Supplementary file 1 [file molecules-28-02898-s001.zip › molecules-2244016-supplementary.docx]

**Table S1.** Concentration of the phenolic compounds found in the studied *A. annua* aqueous extract (AAE) with corresponding Limit of Detection (LOD), and Limit of Quantitation (LOQ) values based on HPLC analysis.

| **Sample** | **Chlorogenic acid (ppm)** | **Caffeic acid (ppm)** |
| --- | --- | --- |
| AAE | 28.64±0.55 | 7.70±0.08 |
| LOD (ppm) | 4.05 | 0.58 |
| LOQ (ppm) | 12.28 | 1.75 |

**Calibration curve of chlorogenic acid**

**Figure S1.** Calibration curve based on HPLC analysis used to quantify chlorogenic acid in *A. annua* aqueous extract (AAE).

Calibration curve of caffeic acid

**Figure S2**. Calibration curve based on HPLC analysis used to quantify caffeic acid in *A. annua* aqueous extract (AAE).
